# Comparative study of rabies antibody titers of dogs vaccinated in Finland and imported street dogs vaccinated abroad

**DOI:** 10.1186/s13028-019-0450-8

**Published:** 2019-03-14

**Authors:** Marianne Kaila, Jasmine Marjoniemi, Tiina Nokireki

**Affiliations:** 1 Virology Unit, Finnish Food Authority, Mustialankatu 3, 00790 Helsinki, Finland; 2 University of Helsinki, P.O. Box 66, 00014 Helsinki, Finland

**Keywords:** Antibody, Canine distemper, Import, Prophylaxis, Rabies, Street dogs, Vaccination

## Abstract

Seventy-two canine serum samples were analyzed for post-vaccination serum titers of rabies antibodies. The samples were divided into two groups: Group 1 dogs (n = 36) were imported dogs from the Russian Federation (n = 31) or Romania (n = 5), with a mean serum antibody titer value of 1.54 IU/mL. Group 2 dogs (n = 36) were Finnish dogs vaccinated in Finland, with a mean titer of 4.19 IU/mL. Altogether, 14 (39%) dogs (CI 95% 23–56) were without detectable antibodies (≤ 0.1 IU/mL) in Group 1, whereas in Group 2, all dogs had an antibody titer greater than 0.1 IU/mL. A statistically significant difference was observed between these groups when comparing the proportions of dogs with antibody levels less than or exceeding 0.5 IU/mL. In Group 1, 19 out of the 36 dogs (CI 95% 36–70) had serum titer values < 0.5 IU/mL, while in Group 2, only 2 dogs had serum titer values < 0.5 IU/mL. Despite the small sample size, this raises concern over the imported dogs having insufficient antibody levels required for international travel and implies that these dogs had perhaps not been vaccinated, even though they had documentation of vaccination upon arrival.

## Findings

Over 60 000 people die each year from rabies, and up to 99% of human cases originate from a dog bite. Rabies is preventable by pre- and/or post-exposure prophylaxis consisting of a series of rabies vaccinations and in some cases by the use of immunoglobulins. Each year, an estimated 7 million people are bitten by suspected rabid dogs. The most cost-effective way to eliminate human rabies is to eliminate rabies in dogs [1].

Rabies is endemic in the Russian Federation. In 2017, there were 1 791 reported cases in animals and three human cases [2]. In Romania, rabies cases in wild foxes (*Vulpes vulpes*) have decreased in recent years due to an oral vaccination program as a part of an eradication program partially supported by the EU. There were two reported cases in 2017 in domestic animals [2]. Finland has been officially rabies free since 1991, even though lyssaviruses have been detected in bats [3–5]. Canine distemper (CD) is very rare in the Finnish dog population due to a recommended vaccination program, and during recent years, only imported cases have been detected [6]. In recent years, the import of street dogs to Finland has dramatically increased. In 2017, 2454 street dogs were imported to Finland compared to only 289 in 2010. Thus, in less than a decade the number of adopted street dogs in Finland has multiplied by a factor of ten. Import of street dogs represent a threat for the spread and introduction of various pathogens such as rabies virus and canine distemper virus (CDV).

Virus-neutralizing antibody assays are used to verify that a humoral immune response has occurred after vaccination against rabies. An internationally accepted threshold titer of 0.5 IU/mL has been adopted [7]. Failure of vaccination may leave the animal susceptible to rabies virus and thus increase the risk developing rabies with an obvious zoonotic risk. Previous studies have demonstrated that the antibody response is influenced by the vaccine product used, the number of vaccine doses, the time between vaccinations and blood sampling, and the age, size, and breed of the dog [8–11]. Studies have suggested a failure of vaccination in imported dogs [12, 13]. Rota Nodari et al. [13] found a higher vaccination failure rate in imported dogs (13.15%) than in those vaccinated in Italy (5.89%) and De Benedictis et al. [14] reported a vaccination failure rate as high as a 37% in dogs crossing the Italian border from Eastern European countries.

The aim of this study was to investigate the rabies antibody levels in street dogs vaccinated in and imported from the Russian Federation or Romania, and to compare the antibody titers with those of dogs vaccinated in Finland. The need to compare these two groups arose due to suspicion of forged importation documents. The serum samples were additionally analyzed for antibodies against CDV since one dog in Group 1 was euthanized due to CD.

The study material comprised serum samples from 72 dogs vaccinated against rabies. These were grouped in two groups: Street dogs imported into Finland and vaccinated in either the Russian Federation (n = 31), or Romania (n = 5) (n = 36, Group 1) and dogs born, raised and vaccinated in Finland (n = 36, Group 2). The samples were taken between January and March 2018. The shortest time from vaccination to sampling was 3 weeks. The age range of the dogs was from 3 months to 16 years. Of all the dogs (n = 72), 54 had only been vaccinated once, while 18 dogs had been vaccinated at least twice. The antibody responses of the dogs were determined using the fluorescent antibody virus neutralization (FAVN) test [15]. CDV antibodies were determined from serum using a seroneutralization test [16]. Samples with a titer of < 1:8 were considered negative. Since the serum samples were submitted to determine the rabies virus antibody level, we had no data on the CDV vaccination status in either group. In Finland, rabies vaccination is recommended for dogs at the age of 4 months and CD vaccination at the age of 3 months with a booster at the age of 4 months.

The Chi squared test was used to analyze the possible difference between proportions of rabies antibodies (< 0.5 IU/mL; ≥ 0.5 IU/mL) and CDV antibodies (< 1:8; ≥ 1:8).

**Fig. 1:**
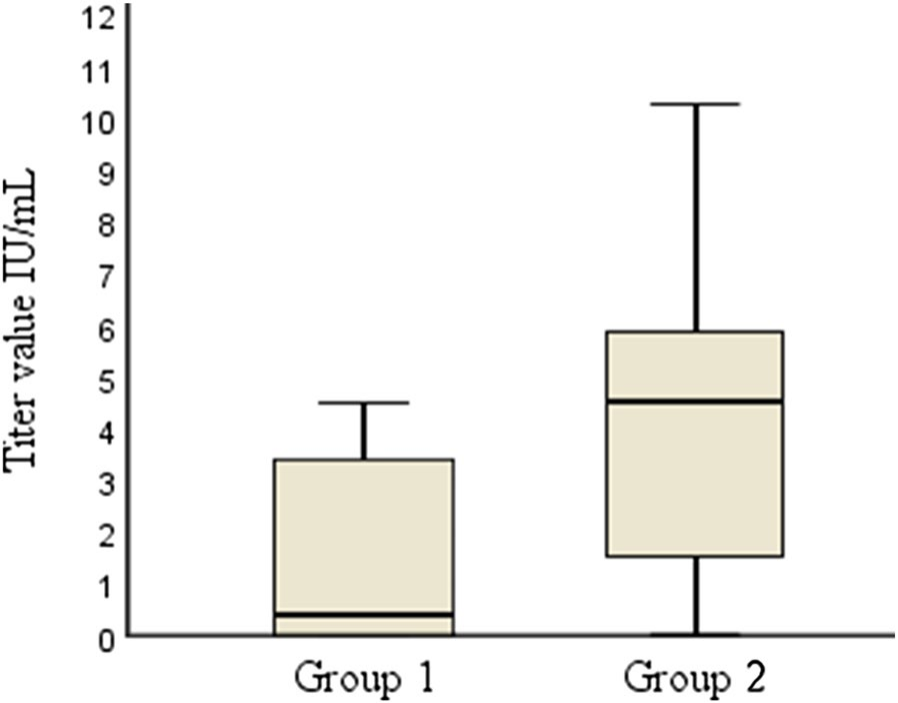
Rabies vaccination titers in dogs vaccinated either in the Russian Federation or Romania (Group 1) or in Finland (Group 2). Median value for Group 1 is 0.35 IU/mL with a value range of 4.5 (0.0–4.5). Median value for Group 2 is 4.5 IU/mL with a value range of 10.20 (0.1–10.30)

In Group 1, the mean titer value was 1.5 IU/mL. Mean titer for dogs from the Russian Federation was 1.62 IU/mL and for Romanian dogs 1.08 IU/mL. Despite the group mean being over the threshold of ≥ 0.5 IU/mL, over half of the dogs (19/36) had an antibody level < 0.5 IU/mL, the mode of the titer value being under the detection limit and the median 0.35 IU/mL. In Group 2, the mean titer value was 4.21 IU/mL. Two dogs (5%) had serum titer values under < 0.5 IU/mL (CI 95% 0–19). All dogs in this group had some immune response, ranging from 0.1 IU/mL up to 10.3 IU/mL. Thirty-four dogs (94%) (CI 95% 81–99) in Group 2 had antibody levels ≥ 0.5 IU/mL (Fig. 1). The findings indicate that a booster vaccination enhanced the immunity in both groups of dogs. Of all the dogs, 54 dogs that had been vaccinated once had a mean titer value of 2.63 IU/mL, while 18 dogs that had been vaccinated at least twice had a mean titer value of 3.63 IU/mL. All the dogs that had received at least one booster vaccination had titer values over 0.5 IU/mL.

Within Group 1, there were 11 (31%) dogs with CDV antibody titer < 1:8 (CI 95% 16–48). In the Group 2, 5 (14%) dogs had a titer value < 1:8 (CI 95% 5–30). The difference between groups was not statistically significant (χ^2^ = 2.89, df = 1, P = 0.089).

According to the legal documents accompanying the imported street dogs (Group 1), all had been vaccinated against rabies at least 21 days before arrival in Finland. However, 19/36 of the Group 1 dogs did not have the internationally accepted serum rabies antibody titer ≥ 0.5 IU/mL. There was a statistically significant difference between Groups 1 and 2 in the proportions of dogs with antibody levels < 0.5 IU/ml and ≥ 0.5 IU/mL. Dogs vaccinated in Finland had a greater probability of having antibody levels ≥ 0.5 IU/mL in comparison to dogs vaccinated in either the Russian Federation or Romania.

Despite taking into consideration factors that may influence antibody development following rabies vaccination such as the time lapse between vaccination and testing, the breed, the size, the age, the type of vaccine and the route of administration [8–11], approximately 90% of vaccinated dogs achieve an adequate immune response after one dosage of rabies vaccine and only around 1% are so-called “non-responders”, which fail to produce antibodies [17]. Although there are several reasons why vaccination does not always lead to sufficient development of antibodies, our finding that 39% of the imported dogs had serum titers < 0.1 IU/mL suggests that at least some of them might not have been vaccinated at all. The rabies vaccines used for the dogs in this study should have provided a satisfactory response in the majority of the dogs if used and stored correctly. A young age of the imported dogs is not the reason for the low titers since only two of the imported dogs were under the age of 4 months at the time of vaccination and both had antibody titer > 0.5 IU/mL. Rota Nodari et al. [13] demonstrated that the discrepancy in vaccine failure rates for dogs sampled > 75 days after vaccination was still significant between dogs vaccinated in Italy and imported dogs, meaning that even when testing > 75 days after vaccination, the vaccination response was truly different between groups.

When people have been bitten by recently imported street dogs, post-exposure prophylaxis is needed, causing considerable costs to the healthcare system and concern for persons who have been bitten. The increased adoption of street dogs from countries where rabies is still prevalent is an alarming trend. The data indicate that the level of compliance with the importation regulation may be low. Even though rabies virus in the Russian Federation is not circulating in the dog population but in wildlife, it should be considered whether testing for rabies vaccination antibodies should be required when importing dogs from rabies endemic countries. Norway has declared that street dogs will no longer be legal to commercially import. This means that it is no longer possible to adopt or import a street dog into Norway as of 1.7.2018. A private person must document that the dog has been owned and lived with the importer for a minimum of 6 months before entering Norway [18].

The data also revealed that some dogs in both groups had an insufficient CDV vaccination response. There was no statistically significant difference between Groups 1 and 2. Since no data were available on the vaccination history of the dogs or previous exposure to CDV, we hypothesize that there were CDV susceptible animals among the imported street dogs as well as among Finnish dogs. Owners should be encouraged to maintain good vaccination coverage in order to prevent future epidemics of CVD. People importing street dogs should acknowledge that the importation of street dogs from rabies-endemic countries into the EU may pose a threat to human health. Even though one rabid dog might not cause an outbreak, the severity of rabies enforces the need of prophylaxis.
